# Microstructure and Tribological Properties of Spark-Plasma-Sintered Ti_3_SiC_2_-Pb-Ag Composites at Elevated Temperatures

**DOI:** 10.3390/ma15041437

**Published:** 2022-02-15

**Authors:** Rui Zhang, Huiming Zhang, Fuyan Liu

**Affiliations:** 1 School of Mechanical Engineering, Chengdu University, Chengdu 610106, China; zhm121998@163.com; 2 Sichuan Province Engineering Technology Research Center of Powder Metallurgy, Chengdu University, Chengdu 610106, China; 3 Institute for Advanced Materials Deformation and Damage from Multi-Scale, Chengdu University, Chengdu 610106, China; 4 School of Mechanical Engineering, Xinjiang University, Urumqi 830000, China; 5 School of Chemical Engineering and Materials, Changzhou Institute of Technology, Changzhou 213032, China

**Keywords:** Ti_3_SiC_2_-PbO-Ag composites, tribo-chemical reaction, self-lubricating composite, tribo-oxidation

## Abstract

Ti_3_SiC_2_-PbO-Ag composites (TSC-PA) were successfully prepared using the spark plasma sintering (SPS) technique. The ingredient and morphology of the as-synthesized composites were elaborately investigated. The tribological properties of the TSC-PA pin sliding against Inconel 718 alloys disk at room temperature (RT) to 800 °C were examined in air. The wear mechanisms were argued elaborately. The results showed that the TSC-PA was mainly composed of Ti_3_SiC_2_, Pb, and Ag. The average friction coefficient of TSC-PA gradually decreased from 0.72 (RT) to 0.3 (800 °C), with the temperature increasing from RT to 800 °C. The wear rate of TSC-PA showed a decreasing trend, with the temperature rising from RT to 800 °C. The wear rate of Inconel 718 exhibited positive wear at RT and negative wear at elevated temperatures. The tribological property of TSC-PA was related to the tribo-chemistry, and the abrasive and adhesive wear.

## 1. Introduction

Ti_3_SiC_2_ belongs to one of the MAX phases (polycrystalline nanolaminates of ternary carbides and nitrides, over 60 + phases) [1]. It possesses the combined characteristics of metal and ceramics, such as being easily machinable, being electrically and thermally conductive, being oxidation resistant, and having a high melting point. Ti_3_SiC_2_ can be a potential candidate for structural functional components. Similar to graphite and MoS_2_, it has a hexagonal structure, endowing it with an exceptional lubricating property. Research on the tribological properties of Ti_3_SiC_2_ demonstrated that it is not a special solid lubricant. Contrarily, Ti_3_SiC_2_ generally has a high friction coefficient (μ > 0.6) and a high wear rate (WR: in the order of 10^−2^–10^−3^ mm^3^/N m), especially at medium and low temperatures (RT) [2,3,4,5,6,7,8]. It has been reported that, in some circumstances (such as with temperature > 600 °C, at a speed of >5 m/s, or sliding against some particular tribopair material), polycrystalline Ti_3_SiC_2_ displays the relatively low μ and wear rate, which mainly results from the tribo-oxidation transfer films generated on the contact surface [2,6,8,9,10]. In other situations, the fracture and pulling-out of Ti_3_SiC_2_ grains causes three-body abrasive wear, which is the main wear mechanisms for Ti_3_SiC_2_.

Subsequently, most of scientific enthusiasts concentrate on the intensifying research of polycrystalline Ti_3_SiC_2_ by uniting metal and/or ceramic to enhance its friction and wear behaviors. Ren et al. [11] prepared Ti_3_SiC_2_/Cu/Al/SiC composites, and they found that the tribological behaviors of the composite were better than those of polycrystalline Ti_3_SiC_2_ at RT and 200 °C, while the wear properties of the composite were worse than that of polycrystalline Ti_3_SiC_2_ at elevated temperatures (≥400 °C). The incorporation of Al, Cu, SiC and Al_2_O_3_ played an important role in reinforcing the bonding of Ti_3_SiC_2_ grains and in fixing the soft Ti_3_SiC_2_ matrix around them during reciprocate sliding at RT–200 °C, where the wear mechanism was abrasive wear. At elevated temperatures (≥400 °C), the plastic flow of tribo-oxides layers led to the higher wear rate of the composite than that of Ti_3_SiC_2_ where the wear mechanism was adhesive wear. Yang et al. [12] prepared Cu-Ti_3_SiC_2_ co-continuous composites. They showed excellent electrical conductivity and wear resistance due to the addition of Cu. Yang et al. [13] explored the tribological behaviors of (TiB_2_ + TiC)/Ti_3_SiC_2_ composites. It was discovered that μ of the composites was bigger compared with that of Ti_3_SiC_2_. The addition of TiB_2_ and TiC could fix the soft matrix around them and scatter the shear stresses. In our earlier work [14], we discussed the tribological behavior of Ti_3_SiC_2_/CaF_2_ composites at elevated temperatures. The prepared Ti_3_SiC_2_/CaF_2_ exhibited better friction and wear property than Ti_3_SiC_2_ in a wide temperature range due to the tribo-oxide competition. Islak et al. [15] investigated the effect of reinforcing TiB_2_ particles (5, 10, and 15 wt.%) on the properties of as-synthesized Ti_3_SiC_2_. The addition of TiB_2_ improved the mechanical properties and thermal diffusivity of the composites. Moreover, the addition of TiB2 significantly increased the wear properties of Ti_3_SiC_2_ matrix. Magnus et al. [16] prepared the Ti_3_SiC_2_-TiSi_2_-TiC composites by spark plasma sintering (SPS). They discovered that the tribological property of the composites was attributed to the intrinsic lubricity and the addition of second phase TiC particles. During the sliding, the tribo-oxidative wear was the main wear mechanism; then, it changed to the deformation-induced wear. The transition resulted from the breakdown of the formed tribofilm.

Silver shows some outstanding characteristics (such as good thermal conductivity, favorable ductility, and special mechanical behaviors) and can be widely applied in many fields, such as air-foil bearings, and biomedical and thermal interface materials. It is commonly utilized as a solid lubricant at temperatures below 500 °C. Additionally, it has a high diffusion coefficient and easily generates a lower shear stress intersection at the sliding interface [17,18]. Thus, it was significant and practical to incorporate good properties of Ag with Ti_3_SiC_2_. The goal of this work was to synthesize Ti_3_SiC_2_/Ag composites and to uncover the mechanical and tribological behaviors of the composite at raising temperatures. F. AlAnazi et al. [19] prepared metals-based (Ag and Bi) composites incorporation with 5 vol%, 10 vol%, 20 vol%, and 30 vol% Ti_3_SiC_2_ and investigated their tribological properties. They found that the incorporation of Ti_3_SiC_2_ improved the hardness and compressive yield strength of the two composites. Additionally, the addition of Ti_3_SiC_2_ enhanced the tribological performance of the two composites. Zeng et al. [20] inspected the tribological behaviors of Ti_3_SiC_2_/Ag composites with Inconel 718 as the tribopair at RT. At different sliding speeds, the composites showed lower μ and wear rate than that of Ti_3_SiC_2_.

Pb is lubricous due to its low shear strength. In our previous work [21,22], we found that the incorporation of PbO (melting point: 888 °C) instead of Pb (melting point: 327 °C) in Ti_3_SiC_2_ can successfully prepare Ti_3_SiC_2_/Pb composites and avoid the loss of Pb during the sintering process. The as-prepared composite showed better friction and wear behaviors than Ti_3_SiC_2_ at elevated temperatures. 

As a result of the above-mentioned facts, it was necessary to suggest some novel composites that can move forward a single step in strengthening the tribological performance of Ti_3_SiC_2_ in a wide temperature range. Therefore, in this work, the metal Pb was added to the Ti_3_SiC_2_/Ag composite to prepare Ti_3_SiC_2_-Pb-Ag composites (TSC-PA). To prevent the loss of Pb during the process of preparing the TSC-PA, PbO was chosen as the source of Pb. The target of this job was to synthesize TSC-PA and to discuss the mechanical and tribological behaviors of the as-synthesized compound at elevated temperatures.

## 2. Experimental Procedure

### 2.1. Materials

The TSC-PA composites were sintered using the powder mixture of Ti_3_SiC_2_, PbO and Ag. The average particle size of Ti_3_SiC_2_ was 3 μm and its purity was ≥98%. It was purchased from Jinhezhi Materials Ltd., Beijing, China. The average particle size of PbO and Ag was 5 μm, and their purity was ≥99%. They were bought from Xilong Chemical Ltd., Shantou, China. The powder composition was 70 vol% Ti_3_SiC_2_-15 vol%PbO-15 vol%Ag. The mixture with a designated composition was blended by ball mill for 6 h. Next, it was loaded in a graphite die. In the end, it was sintered by SPS furnace (Shanghai Chenhua Electric Furnace Co., Ltd., Shanghai, China) in vacuum at 1130 ℃.

### 2.2. Mechanical Properties

The densities of the composites were determined using Archimedes’s principle. Micro-hardness was measured on a Micro-hardness Tester (MH-5-VM, Shanghai Hengyi Technology Co., Ltd., Shanghai, China). with a load of 500 g and a dwell time of 10 s. Flexural strength and compression strength were measured using a universal material tester (SANS-CMT5205, Shenzhen New Sansi Material Testing Co., Ltd., Shenzhen, China). The three-point bending test was conducted to obtain the flexural strength, and the samples were cut to 3 mm high × 4 mm wide × 20 mm long. The cross-head speed and span were 0.05 mm/min and 16 mm, respectively. The sizes of the samples for the compression strength test were Φ 5 mm × 12.5 mm, and the cross-head speed was 0.2 mm/min.

### 2.3. Friction and Wear Test

The friction and wear experiments were carried out in air on a high-temperature tribometer with a pin-on-disk setup (THT01-04015, CSM Instruments SA, Peseux, Switzerland). The TSC-PA was used as a pin, the size of which was Φ 6 mm × 12 mm. The Inconel 718 was used as a disk, and its size was Φ 32 mm × 8 mm. The surfaces of the disk and pin were ground and polished into a surface roughness (Ra) of 0.06 μm. The tribopairs slid against each other for 200 m at a sliding speed of 0.1 m/s under a normal load of 5 N. The friction coefficient was automatically documented by computer during the experiment. The wear volume was obtained by quantifying the volume loss of TSC-PA pin by optical microscopy and by examining the cross-sectional area of the worn area of Inconel 718 disk by 3D surface profilometry (NanoMap-D, Columbus, OH, USA). The wear rates were acquired by Equation (1).

(1) $$\mathrm{Wear}\;\mathrm{rate}\;\left(\frac{{\mathrm{mm}}^{3}}{N\cdot m}\right)=\frac{Wear\;volume\;\left({\mathrm{mm}}^{3}\right)}{Sliding\;distance\;\left(m\right)\times Normal\;load\;\left(N\right)}$$

### 2.4. Analysis

The morphology of TSC-PA was investigated by scanning electron microscopy (SEM, JSM-5600LV, JEOL, Tokyo, Japan) equipped with energy dispersive X-ray spectroscopy. The EDS (IE250, Oxford Instrument, Abingdon, Oxfordshire, UK) was equipped with X-Max Silicon Drift Detector (SDD; the spectral resolution of 124 eV at Mn Kα) and an ultra-thin window; the active area of the SDD was 50 mm^2^. The phase composition of TSC-PA was analyzed by X-ray diffraction (XRD, Philips X’Pert Pro, PANalytical Netherlands, Almelo, Netherland) using Cu Kα radiation (λ = 0.15418 nm) within the 2θ-angle range from 5° to 90°. X-ray photoelectron spectroscope (XPS, PHI-5702, Physical Electronics Corporation, Chanhassen, MN, USA) was used to analyze the elemental chemical states of TSC-PA on the worn surfaces, and the binding energy of adventitious carbon (C1s: 284.8 eV) was used as the reference.

## 3. Result and Discussion

### 3.1. Phase Composition and Microstructure of TSC-PA

**Figure 1:**
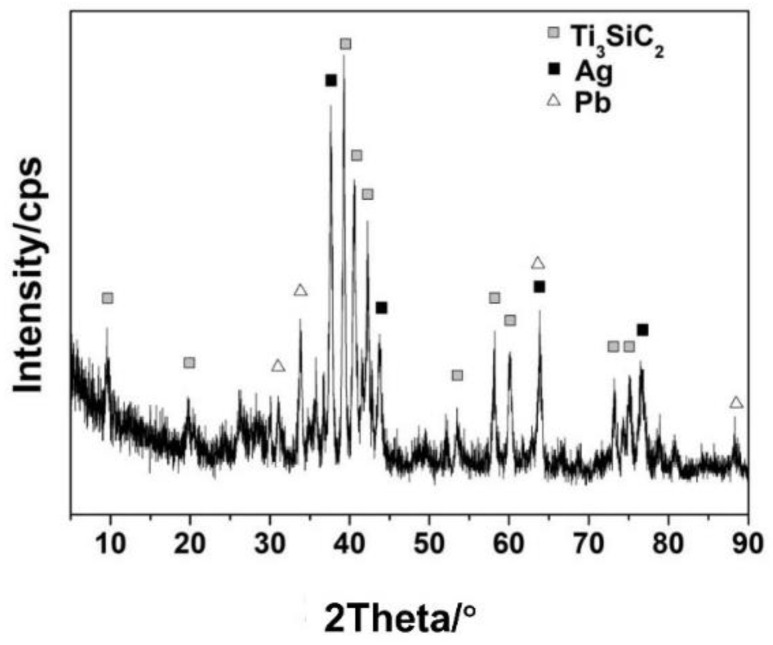
The XRD pattern of TSC-PA.

The XRD pattern of TSC-PA is displayed in Figure 1. As noticed in Figure 1, the main phases of TSC-PA were Ti_3_SiC_2_, Pb, and Ag. The Ti_3_SiC_2_ was temporarily stable and did not break down during the sintering process of TSC-PA. To the best of the authors’ knowledge, the Ti_3_SiC_2_ revealed high reaction activity when in contact with the metal phases, such as Cu [23,24,25], Al [26,27], and Fe [28]. In this study, the Ti_3_SiC_2_ did not decompose with the co-addition of Ag and PbO at the sintering temperature of 1130 °C. In a previous paper [21], we reported that PbO in the Ti_3_SiC_2_-PbO system was deoxidized to Pb by C or Si during the preparation process and Ti_3_SiC_2_ did not decompose at 1200 °C. The relevant chemical reactions were described in Reference [21]. In comparison, the addition of Ag did not change the composition of the Ti_3_SiC_2_-PbO system.

**Figure 2:**
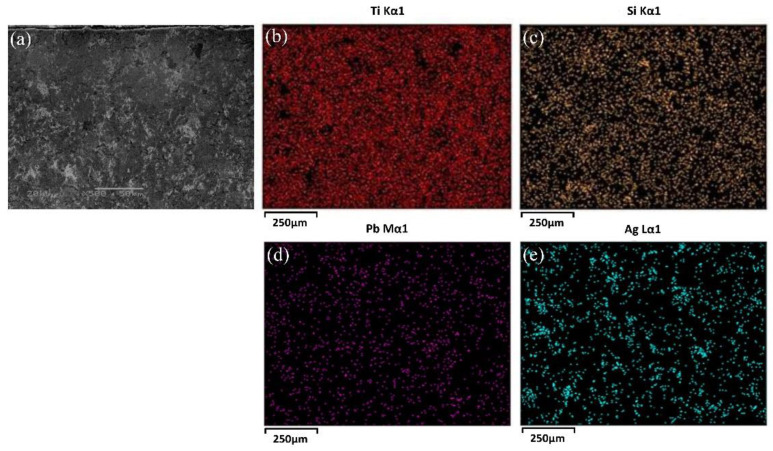
SEM micrograph (**a**) and distribution of Ti (**b**), Si (**c**), Pb (**d**), Ag (**e**) elements in the cross section of TSC-PA.

As observed in Figure 2, the morphology and the distributions of elements of the cross section of the TSC-PA was analyzed using SEM. It can be observed that the Pb particles were homogeneously scattered in the Ti_3_SiC_2_ matrix, while the Ag particles were agglomerated to a certain extent. As the melting point of silver was merely 961 °C, some amount of molten silver nearby flowed together during sintering. Therefore, the agglomeration of Ag occurred in the TSC-PA.

### 3.2. Mechanical Performance of TSC-PA

The relative density, microhardness, flexural strength, and compressive strength of TSC-PA and TSC are listed in Table 1. It can be seen that the relative densities of TSC-PA and TSC were 95.35% and 98.24%, respectively. The microhardness of TSC-PA was less than half that of TSC, and their microhardness values were 2.24 and 5.5 GPa, respectively. Pb and Ag in the TSC-PA belong to soft metals and caused the low hardness of TSC-PA. The flexural strength of TSC-PA was less than that of TSC, and their values were 311 and 428 MPa, respectively. The compressive strength of TSC-PA was only half that of TSC, and their values were 654 MPa and 1230 MPa, respectively. 

**Figure 3:**
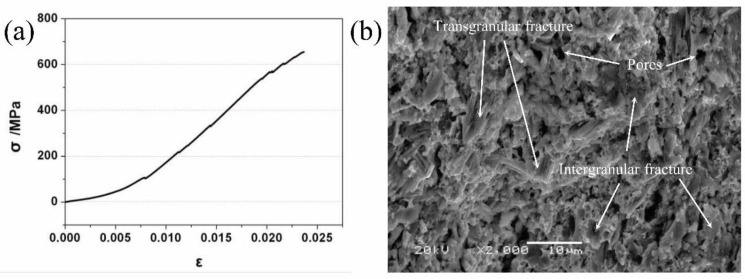
(**a**) Compressive stress–strain curve of TSC-PA and (**b**) SEM image of the fractured surface of TSC-PA after the three-point bending test.

As shown in Figure 3a, the TSC-PA only suffered from elastic deformation before fracture, which was a typical brittle fracture. The fracture surface of TSC-PA (see Figure 3b) indicated that the fracture surface of TSC-PA was featured by intergranular and transgranular fracture. This fracture mode of TSC-PA was similar to TSC. As well known, the diverse energy-absorbing strategies including the diffuse of the microcracks, lamination, crack deflection, and grain pull-out were discovered to be in charge of the high flexural strength of TSC [29,30]. In addition to intergranular and transgranular fracture, some pores and cracks resulting from the unsatisfactory compatibility (e.g., wettability) of the TSC matrix and Pb and Ag were observed (see Figure 3b). The thermal expansion coefficients (for instance: TSC: 9.1 × 10^−6^, Pb: 29.3 × 10^−6^, and Ag: 19.5 × 10^−6^ °C^−1^) of different phases were not matched with each other, leading to the weak bonding between granules and the formation of some defects (such as micro-pores and micro-cracks) during the preparation procedure. The as-formed defects were detrimental to the flexural strength. An additional major justification for the poor fracture strength of the TSC-PA was that the distribution of Pb and Ag around the TSC grains to a certain extent hindered the diverse energy-assimilating strategies, which let fracture take place between the grains. 

### 3.3. Tribological Behavior of TSC-PA

**Figure 4:**
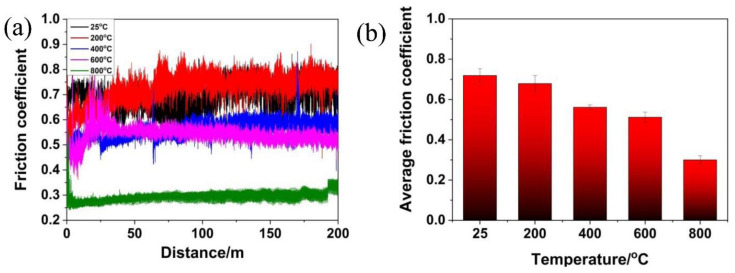
Plot of (**a**) friction coefficient (μ) versus distance and (**b**) the average friction coefficients of TSC-PA sliding against Inconel 718 alloys with different temperature.

Figure 4a showed the plot of μ versus distance curve of the TSC-PA/Inconel 718 tribopair at elevated temperatures. It can be easily construed that the temperature had a direct effect on the μ of the tribopair. At RT and 200 °C, the μ had a big fluctuation. At 400, 600, and 800 °C, the incipient μ had a big fluctuation and, then, it soon achieved a steady state with low μ values. Specifically, at RT, the μ showed a larger fluctuation than that at other temperatures. Particularly, at 600 °C, the μ exhibited a turbulent behavior in contrasted with that at other temperatures, which indicated an incipient short break-in period with relatively low μ values; then, it reached high μ values and fluctuated largely. Later, it approached a stable state with relatively low μ values. In comparison, the fluctuation of the μ decreased with the temperature increasing from RT to 800 °C. Figure 4b compared the μ_mean_ of TSC-PA/Inconel 718 tribopair at elevated temperatures. It can be seen that the μ_mean_ decreased with the temperature increasing from RT to 800 °C.

**Figure 5:**
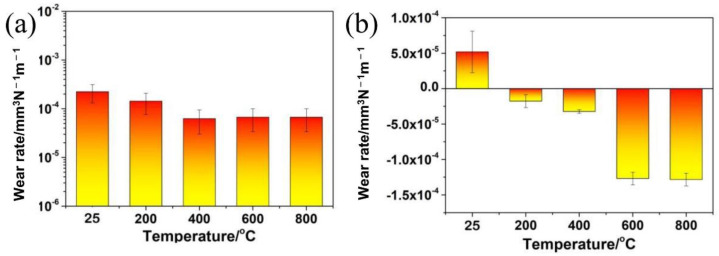
The wear rates of (**a**) pin and (**b**) disk for TSC—PA/Inconel 718 alloy tribo-pair as a function of temperature.

Figure 5a plotted the wear rates of the TSC-PA pin at elevated temperatures. The wear rates of the pin decreased with the temperature increasing from RT to 400 °C. Moreover, the wear rates of the pin were the same when the temperature was at 400, 600, and 800 °C. No other results were found in the literature for a comparison with the results in this work because Ti_3_SiC_2_ composites with the co-addition of PbO and Ag have not been reported before. Moreover, it was not easy to make a comparison between tribology results as they were carried out under distinct conditions on the basis of the detailed demands of an application. For interpretative aspirations, Table 2 compared tribological behavior of Ti_3_SiC_2_ composites with the addition of the different amount of Ag or the addition of the different amount of PbO. Comparatively, the incorporation of relative low content (5–20 vol%) of Ti_3_SiC_2_ can diminish the wear rate of the Ag-based composite during sliding against Al_2_O_3_ at 5N and 0.5 m/s [19]. In this work, the situation was different because the sample was Ti_3_SiC_2_ based-composite with 70 vol% of Ti_3_SiC_2_. At RT, comparatively, Ti_3_SiC_2_ and composites of Ti_3_SiC_2_ with 15 vol% Ag, with 15 vol% PbO, and with 15 vol% PbO-15 vol% Ag showed wear rates of 2 × 10^−3^ mm^3^/(N·m), 3 × 10^−5^ mm^3^/(N·m), 2 × 10^−4^ mm^3^/(N·m), and 2 × 10^−4^ mm^3^/(N·m), respectively, during sliding against Inconel 718 at 5N and 0.1 m/s [20,22]. These results showed that the incorporation of 15 vol% Ag or 15 vol% PbO or 15 vol% PbO-15 vol% Ag can decrease the wear rate of Ti_3_SiC_2_ by at least an order of magnitude at RT. At elevated temperatures (>200 °C), the composite of Ti_3_SiC_2_ with 15 vol% PbO showed better wear resistance than both Ti_3_SiC_2_ and the composite of Ti_3_SiC_2_ with 15 vol% PbO-15 vol% Ag (TSC-PA). The tribological behavior of the composite of Ti_3_SiC_2_ with 15 vol% Ag at elevated temperature was unavailable and incomparable. It can be seen from Table 2 that TSC-PA showed a lower friction than TSC at > 200 °C and a lower friction than Ti_3_SiC_2_-15 vol% PbO at <600 °C. It was concluded that the addition of PbO and Ag apparently improved the tribological behavior of TSC-PA, in comparison with TSC and Ti_3_SiC_2_-15 vol% PbO.

Figure 5b compared the wear rates of the Inconel 718 disk sliding against TSC-PA at elevated temperatures. As the temperature raised from RT to 800 °C, the wear rates of the disk decreased. At RT, a positive wear rate was found for the disk. Interestingly, negative wear rates were detected for the disk and increased with the temperature rising from 200 to 800 °C. Moreover, the wear rate of the disk at 600 °C was identical to that at 800 °C. The detection of Ti, Si, Pb, and Ag on the worn surface of Inconel 718 was in line with the negative wear of the disk (see Table 3). 

### 3.4. Tribological Mechanisms

**Figure 6:**
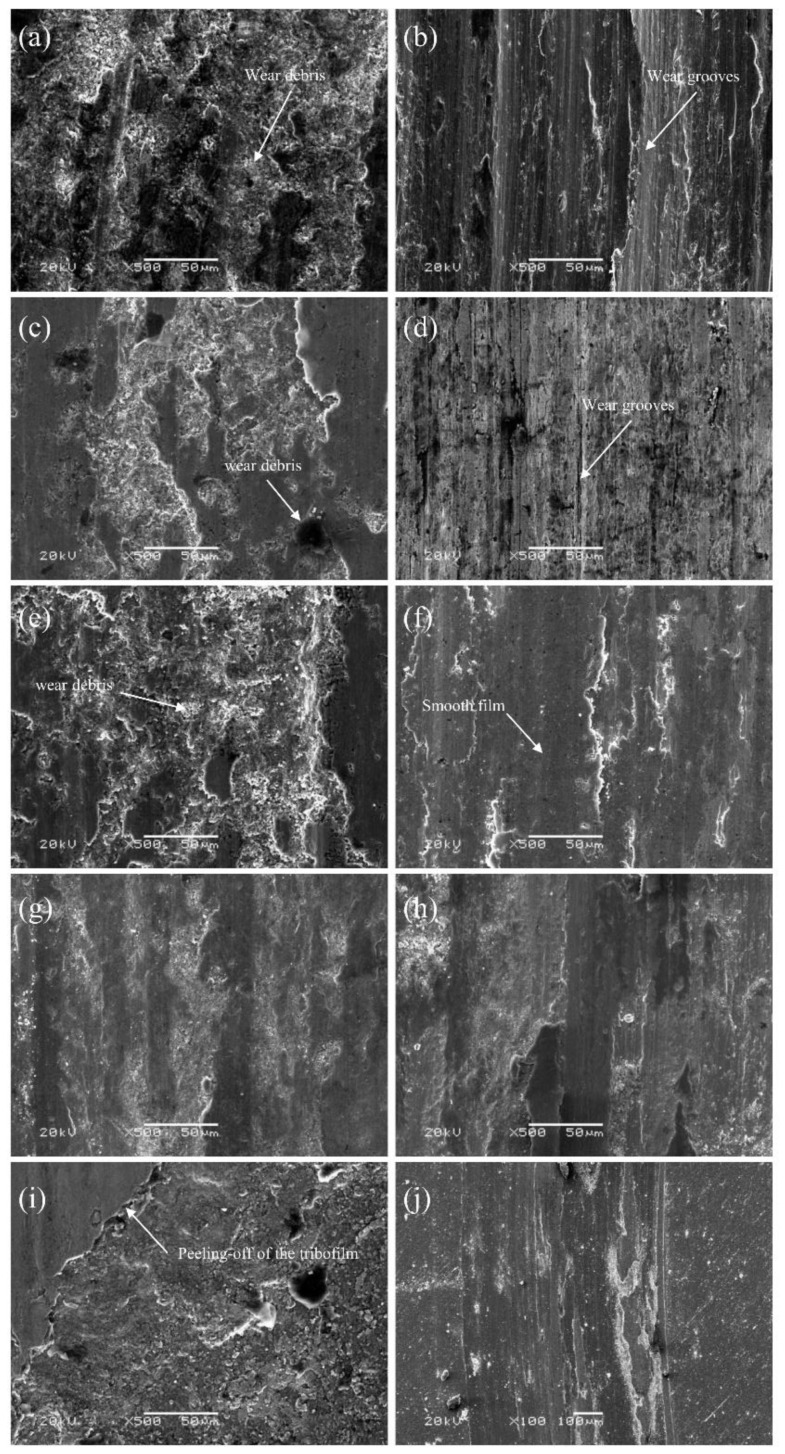
SEM micrographs of (**a**) TSC-PA at 25 °C, (**b**) Inconel 718 alloy at 25 °C, (**c**) TSC-PA at 200 °C, (**d**) Inconel 718 alloy at 200 °C, (**e**) TSC-PA at 400 °C, (**f**) Inconel 718 alloy at 400 °C, (**g**) TSC-PA at 600 °C, (**h**) Inconel 718 alloy at 600 °C, (**i**) TSC-PA at 800 °C, and (**j**) Inconel 718 alloy at 800 °C after tribological testing.

**Figure 7:**
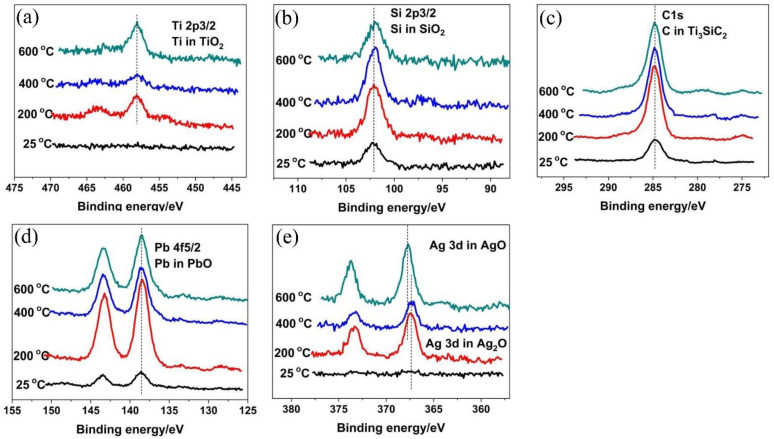
X-ray photoelectron spectroscopy for (**a**) Ti2p, (**b**) Si2pc, (**c**) Pb4f, (**d**) Ag3d, and (**e**) C1s on the worn surface of TSC-PA after sliding against Inconel 718 alloy with different temperature.

Figure 6 showed the SEM assessment of tribosurfaces of TSC-PA/Inconel 718 tribocouple after tribological testing at elevated temperature. TSC-PA was full of scars resulting from the abrasive wear, while the Inconel 718 surface was covered with an even tribolayer delivered from the TSC-PA surface. The surface was surrounded by the tribolayer made up of incompletely oxidized TSC-PA (Table 3 and Figure 6). With the rise in temperature, the tribofilm formed on the surface of TSC-PA was smoother and denser. This film was formed undoubtably by a coalescence of diverse mechanical blending, fracturing, resintering, and mechanically vitalized oxidation and other chemical processes, perhaps, continuously refined during the heat cycle. The EDS data showed that the elements of Ni, Cr, and Fe from Inconel 718 alloy appeared on the surface of TSC-PA (Figure 6a,c,e,g,i in Table 3) and the elements of Ti, Si, Pb, and Ag from TSC-PA appeared on the surface of Inconel718 alloy (Figure 6b,d,f,h,j in Table 3). Moreover, tribo-oxidation was a predominant factor that influenced, and even controlled, the wear mechanism and finally brought about the change of friction and wear features [31]. The chemical states of Ti, Si, Pb, and Ag elements on the worn surface of TSC-PA at RT–800 °C were investigated in detail by XPS (see Figure 7).

At RT, some wear debris dispersed on the worn surface of the TSC-PA with several dark continent regions (see Figure 6a). It was worth mentioning that some loose cracks and pores scattered on the worn surface, which was due to the peeling-off and decentralization of the granules. Such an episode was in accordance with Ti_3_SiC_2_ [6]. Therefore, it can be deduced that the addition of Pb and Ag did not affect the wear behavior of Ti_3_SiC_2_ at RT. Though the weak bonding between Ti_3_SiC_2_ grains in the TSC-PA led to the fracture and pulling out of Ti_3_SiC_2_ granules, soft metals (including Pb and Ag) distributed around the Ti_3_SiC_2_ grains, inhibiting the peeling-off and decentralization of Ti_3_SiC_2_ matrix; therefore, the wear rate of TSC-PA was only one tenth of Ti_3_SiC_2_ (see Table 2). A third body was formed and entrapped between the pin and disk, leading to the severe wear of TSC-PA and Inconel 718 at RT. The obvious evidence was that wear grooves were found on the worn surface of Inconel 718 after tribology testing at RT (see Figure 6b) and mutual material transfer between the pin and disk was detected by EDS analysis (see Table 3). The abrasive wear was the primary wear mechanism for TSC-PA. A small amount of SiO_2_ and PbO was detected on the worn surface of TSC-PA (see Figure 7). However, the temperature was too low to form an enough thick tribo-oxidation film between the contact surface of TSC-PA and Inconel 718, so high friction coefficients and high wear rates were present at RT (see Figure 4 and Figure 5a).

At 200 °C, some compacted wear debris was found on the worn surface of TSC-PA (see Figure 6c). Compared with RT, besides SiO_2_ and PbO, other oxides, TiO_2_ and Ag_2_O, were formed on the surface of TSC-PA (evidently owing to the XPS at 200 °C in Figure 7), generating a thicker tribo-oxidation film. The formation of the tribo-oxidation film did not inhibit the fracture and pulling-out of Ti_3_SiC_2_ grains. The formation and entrapment of the third body (the formed wear debris) between the pin and disk contributed to the wear of TSC-PA and Inconel 718. The mutual material transfer between the pin and disk at 200 °C was detected by EDS analysis (see Table 3). A tribo-oxidation film with some wear grooves was transferred to the Inconel 718, so the counterpart showed relatively flat region and slight negative wear (see Figure 5b and Figure 6d). At 200 °C, the abrasive wear mechanism was the main wear mechanism with very slight adhesive wear. 

At 400 °C, obviously, the TSC-PA was covered with some large wear debris after tribology testing (see Figure 6e). It was expected that, initially, a tribo-oxidation film containing TiO_2_, SiO_2_, PbO and Ag_2_O was formed on its surface (as evident owing to the XPS at 400 °C in Figure 7), which was similar to the oxidation composition at 200 °C. Then, the peel-off and removal of the tribo-oxidation film occurred for TSC-PA. As the sliding finished, the mutual material transfer between the pin and disk was detected by EDS analysis (see Table 3). It was seen from Figure 6f that a relatively smooth film was built up on the surface of the Inconel 718. The plastic flow, in some way, retarded the abrasive wear of TSC-PA; therefore, the wear rate of TSC-PA at 400 °C exhibited a distinct decline in contrast to those at RT and 200 °C. Moreover, the addition of Ag and Pb could strengthen the ductility of TSC-PA. On other hand, when the asperities of the tribo-pair were in contact with each other, the instant flash temperature may reach 3000 °C [32]. Therefore, a high temperature was beneficial for the formation of oxides, such as TiO_2_, SiO_2_, PbO, and Ag_2_O. Therefore, the adhesive wear and plastic flow was the main wear mechanism with slight abrasive wear (as distinct on account of the slight wear trenches on the Inconel 718 disk). 

At 600 °C, the worn surface of TSC-PA was surrounded by a relatively smooth and continuous tribolayer after tribology testing (see Figure 6g). This surface was covered with a sufficiently thick tribo-oxidation film containing TiO_2_, SiO_2_, PbO, and AgO (see Figure 7). In comparison with 400 °C, AgO was found on the worn surface of TSC-PA at 600 °C instead of Ag_2_O. Interestingly, the existence of AgO played a significant role in the decrease of friction and wear of the tribopair. The worn surface of Inconel 718 was embraced by a tribo-oxidation film, which was transferred from TSC-PA (see Figure 6h). The serious plastic flowing resulted in the adhesive wear. Mutual transfer of matter between the pin and the disk was verified by the EDS results in Table 3. Moreover, with the increase in temperature, especially at above 400 °C, oxygen contents of the pin and the disk were higher, which indicated the thicker thickness of the tribo-oxidation film (see Table 3). Therefore, the friction coefficient of TSC-PA was lower and more stable and its wear rate was low due to the moderately thick oxides. The wear mechanism was adhesive wear at this temperature.

At 800 °C, the worn surface of TSC-PA was surrounded by a tribo-oxidation film, which was smoothest and densest among all temperatures (see Figure 6). With the thickness of the tribofilm increasing, the covering of the Inconel 718 surface increased. Matter accumulation on the Inconel 718 led to seizure of the contact. The tribofilm reached a critical thickness so that it was easily peeled off from TSC-PA (see Figure 6i). The Inconel 718 showed a smooth tribofilm with large negative wear (see Figure 5b and Figure 6j). Of course, mutual transfer of matter between the pin and the disk was also confirmed by the EDS data in Table 3. The wear mechanism was adhesive wear and tribochemistry of elements of Pb, Ag, Ti, and Si to form TiO_2_, SiO_2_, PbO, and AgO (see Figure 7). 

Conclusively at RT and 200 °C, the abrasive wear ascribed to the peeling-off and pulled-out of Ti_3_SiC_2_ grains dominated the wear mechanism of TSC-PA, leading to the indispensable friction and wear. As the temperature increased, the formation of tribo-oxidation films was favorable for the reduction in friction and wear of the tribopair. As for the reason why the Inconel 718 disk showed negative wear at elevated temperatures. We proposed that with the temperature increasing, especially at 800 °C, the formed oxidation film was so thick that it was easily peeled-off from the TSC-PA pin and transferred to the Inconel 718 disk, leading to the negative wear of the disk. Additionally, the transition of wear mechanism from abrasive wear to adhesive wear occurred with the increasing temperature. The co-addition of PbO and Ag worked together in the improvement of the tribological behavior of TSC-PA at elevated temperatures, which shed light on the effectiveness of co-addition of solid lubricants in MAX phases at elevated temperatures.

## 4. Conclusions

The Ti_3_SiC_2_-Pb-Ag self-lubricating composite (TSC-PA) was successfully sintered by spark plasma sintering technique in vacuum, and its friction and wear behavior were investigated by sliding it against Inconel 718 using a pin-on disk setup at RT and elevated temperatures in air. The results found that the TSC-PA was mainly composed of Ti_3_SiC_2_, Pb, and Ag. The mechanical properties of the TSC-PA were relatively weaker than that of TSC monolithic. As the temperature increased, the average friction coefficient of TSC-PA/Inconel 718 decreased. The wear rate of TSC-PA exhibited a downward trend and that of Inconel 718 decreased, too, with the rise in the temperature. Interestingly, the wear rate of Inconel 718 was positive wear only at RT and changed to negative wear at other temperatures. The wear mechanism was abrasive wear at low and medium temperatures and turned to adhesive wear at higher and elevated temperatures. The tribochemistry of the sliding contact plays an important part in the transition of the wear theories. It was an effective method of the co-addition of solid lubricants in MAX phases.

## Figures and Tables

**Table 1:** Properties of TSC and TSC-PA.

Sample	No.	Relative Density /%	Microhardness /GPa	Flexural Strength /MPa	Compressive Strength /MPa
Ti_3_SiC_2_ (1250 °C)	TSC	98.24	5.5 ± 0.2	428 ± 10	1230 ± 13
Ti_3_SiC_2_-PbO-Ag	TSC-PA	95.35	2.24 ± 0.14	311 ± 22	654

Note: The data in the first line were from Reference [21].

**Table 2:** Comparison of tribological behavior of different Ti_3_SiC_2_-based composites.

Composition	Counter -Surface	Conditions	Wear rate (mm^3^/Nm)	μ	Reference
Ag-5 vol % Ti_3_SiC_2_	Al_2_O_3_	Block (Tab)-on-Disc, 5N, 0.5 m/s, air, RT	3.2 × 10^−5^	0.38	[19]
Ag-10 vol % Ti_3_SiC_2_		Block (Tab)-on-Disc, 5N, 0.5 m/s, air, RT	2.9 × 10^−5^	0.35	
Ag-20 vol % Ti_3_SiC_2_		Block (Tab)-on-Disc, 5N, 0.5 m/s, air, RT	4.1 × 10^−6^	0.29	
Ag-30 vol % Ti_3_SiC_2_		Block (Tab)-on-Disc, 5N, 0.5 m/s, air, RT	2.5 × 10^−5^	0.3	
Ag		Block (Tab)-on-Disc, 5N, 0.5 m/s, air, RT	4.9 × 10^−5^	0.38	
Ti_3_SiC_2_	Inconel 718	Pin-on-disk, 5N, 0.01 m/s, air, RT	4.0 × 10^−3^	0.62	[20]
		Pin-on-disk, 5N, 0.1 m/s, air, RT	2.0 × 10^−3^	0.61	
		Pin-on-disk, 5N, 1 m/s, air, RT	6.0 × 10^−3^	0.57	
Ti_3_SiC_2_-5 vol% Ag		Pin-on-disk, 5N, 0.01 m/s, air, RT	7.0 × 10^−5^	0.42	
		Pin-on-disk, 5N, 0.1 m/s, air, RT	8.0 × 10^−5^	0.51	
		Pin-on-disk, 5N, 1 m/s, air, RT	5.0 × 10^−3^	0.56	
Ti_3_SiC_2_-10 vol% Ag		Pin-on-disk, 5N, 0.01 m/s, air, RT	4.0 × 10^−5^	0.4	
		Pin-on-disk, 5N, 0.1 m/s, air, RT	7.0 × 10^−6^	0.51	
		Pin-on-disk, 5N, 1 m/s, air, RT	3.0 × 10^−3^	0.5	
Ti_3_SiC_2_-15 vol% Ag		Pin-on-disk, 5N, 0.01 m/s, air, RT	5.0 × 10^−5^	0.4	
		Pin-on-disk, 5N, 0.1 m/s, air, RT	3.0 × 10^−5^	0.5	
		Pin-on-disk, 5N, 1 m/s, air, RT	2.0 × 10^−5^	0.45	
Ti_3_SiC_2_-20 vol% Ag		Pin-on-disk, 5N, 0.01 m/s, air, RT	2.0 × 10^−5^	0.31	
		Pin-on-disk, 5N, 0.1 m/s, air, RT	2.0 × 10^−4^	0.45	
		Pin-on-disk, 5N, 1 m/s, air, RT	1.0 × 10^−5^	0.4	
Ti_3_SiC_2_	Inconel 718 disk	Pin-on-disk, 5N, 0.1 m/s, air, RT	2.0 × 10^−3^	0.65	[22]
		Pin-on-disk, 5N, 0.1 m/s, air, 200 °C	3.0 × 10^−4^	0.65	
		Pin-on-disk, 5N, 0.1 m/s, air, 400 °C	5.0 × 10^−5^	0.62	
		Pin-on-disk, 5N, 0.1 m/s, air, 600 °C	2.5 × 10^−5^	0.65	
		Pin-on-disk, 5N, 0.1 m/s, air, 800 °C	5.0 × 10^−6^	0.4	
Ti_3_SiC_2_-5 vol% PbO	Inconel 718 disk	Pin-on-disk, 5N, 0.1 m/s, air, RT	2.0 × 10^−3^	0.72	
		Pin-on-disk, 5N, 0.1 m/s, air, 200 °C	1.5 × 10^−3^	0.66	
		Pin-on-disk, 5N, 0.1 m/s, air, 400 °C	9.0 × 10^−5^	0.65	
		Pin-on-disk, 5N, 0.1 m/s, air, 600 °C	4.0 × 10^−5^	0.58	
		Pin-on-disk, 5N, 0.1 m/s, air, 800 °C	7.0 × 10^−6^	0.45	
Ti_3_SiC_2_-10 vol% PbO	Inconel 718 disk	Pin-on-disk, 5N, 0.1 m/s, air, RT	6.0 × 10^−4^	0.67	
		Pin-on-disk, 5N, 0.1 m/s, air, 200 °C	6.0 × 10^−4^	0.65	
		Pin-on-disk, 5N, 0.1 m/s, air, 400 °C	3.0 × 10^−5^	0.66	
		Pin-on-disk, 5N, 0.1 m/s, air, 600 °C	1.0 × 10^−5^	0.46	
		Pin-on-disk, 5N, 0.1 m/s, air, 800 °C	5.0 × 10^−6^	0.3	
Ti_3_SiC_2_-15 vol% PbO	Inconel 718 disk	Pin-on-disk, 5N, 0.1 m/s, air, RT	2.0 × 10^−4^	0.71	
		Pin-on-disk, 5N, 0.1 m/s, air, 200 °C	2.0 × 10^−4^	0.75	
		Pin-on-disk, 5N, 0.1 m/s, air, 400 °C	1.0 × 10^−5^	0.65	
		Pin-on-disk, 5N, 0.1 m/s, air, 600 °C	5.0 × 10^−6^	0.35	
		Pin-on-disk, 5N, 0.1 m/s, air, 800 °C	2.0 × 10^−6^	0.2	
Ti_3_SiC_2_-15 vol%PbO-15 vol % Ag	Inconel 718 disk	Pin-on-disk, 5N, 0.1 m/s, air, RT	2.0 × 10^−4^	0.72	This work
		Pin-on-disk, 5N, 0.1 m/s, air, 200 °C	1.5 × 10^−4^	0.67	
		Pin-on-disk, 5N, 0.1 m/s, air, 400 °C	6.0 × 10^−5^	0.56	
		Pin-on-disk, 5N, 0.1 m/s, air, 600 °C	6.0 × 10^−5^	0.51	
		Pin-on-disk, 5N, 0.1 m/s, air, 800 °C	6.0 × 10^−5^	0.3	

**Table 3:** The chemical components of samples corresponding to Figure 6 as determined by EDS.

Positions	Samples	Temperature/°C	Atomic Percentage/at. %
Figure 5a	TSC-PA pin	25	14.9%Ti, 5.2%Si, 29.9%C, 1.4%Al, 1.9%Ag, 42.8%O, 1.0%Pb, 1.3%Ni, 0.7%Cr, 0.9%Fe
Figure 5b	Inconel 718 disk	25	2.4%Ti, 1.3%Si, 28.8%C, 0.9%Al, 0.4%Ag, 25.4%O, 21.0%Ni, 9.4%Cr, 8.4%Fe, 1.3%Nb, 0.7%S
Figure 6c	TSC-PA pin	200	17.3%Ti, 6.3%Si, 31.2%C, 1.3%Al, 1.6%Ag, 39.5%O, 1.0%Pb, 0.8%Ni, 0.5%Cr, 0.5%Fe
Figure 6d	Inconel 718 disk	200	6.5%Ti, 3.3%Si, 31.7%C, 1.4%Al, 0.9%Ag, 33.6%O, 0.5%Pb, 11.1%Ni, 5.3%Cr, 4.6%Fe, 0.7%Nb, 0.4%S
Figure 6e	TSC-PA pin	400	20.5%Ti, 6.1%Si, 23.4%C, 2.0%Al, 2.0%Ag, 42.0%O, 1.2%Pb, 1.5%Ni, 0.6%Cr, 0.7%Fe
Figure 6f	Inconel 718 disk	400	13.0%Ti, 5.7%Si, 18.2%C, 1.9%Al, 2.0%Ag, 46.4%O, 1.0%Pb, 6.1%Ni, 3.0%Cr, 2.7%Fe
Figure 6g	TSC-PA pin	600	9.5%Ti, 3.0%Si, 11.0%C, 0.8%Al, 0.5%Ag, 61.0%O, 0.4%Pb, 7.4%Ni, 3.2%Cr, 2.7%Fe, 0.5%Nb
Figure 6h	Inconel 718 disk	600	2.3%Ti, 0.7%Si, 15.4%C, 0.5%Al, 45.3%O, 1.1%Pb, 17.9%Ni, 7.8%Cr, 6.9%Fe, 1.3%Nb, 0.8%S
Figure 6i	TSC-PA pin	800	9.7%Ti, 2.9%Si, 10.7%C, 0.9%Al, 0.7%Ag, 59.7%O, 0.6%Pb, 7.8%Ni, 3.5%Cr, 2.9%Fe, 0.6%Nb
Figure 6j	Inconel 718 disk	800	11.9%Ti, 5.9%Si, 18.7%C, 1.7%Al, 2.3%Ag, 46.9%O, 0.9%Pb, 5.9%Ni, 3.2%Cr, 2.6%Fe

## Data Availability

The data presented in this study are available within the article.
